# Effects of Biochar and Straw Application on the Physicochemical and Biological Properties of Paddy Soils in Northeast China

**DOI:** 10.1038/s41598-019-52978-w

**Published:** 2019-11-11

**Authors:** Yu Zheng, Xiaori Han, Yuying Li, Jinfeng Yang, Na Li, Ning An

**Affiliations:** 1 College of Land and Environment, Shenyang Agricultural University, Shengyang, 110866 China; 2 Institute of Soil Fertilizer and Environmental Resources, Heilongjiang Academy of Agricultural Sciences, Harbin, 150086 China

**Keywords:** Agroecology, Environmental impact

## Abstract

Applying biochar to soil has been proposed as a strategy to enhance soil quality and crop productivity. To further evaluate the influence of biochar and straw application on soil fertility and crop yield, a five-year fixed site field experiment was conducted in a paddy field in Northeast China. The experimental design included six treatments: control (CK), biochar (C), straw (S), chemical fertilizers (NPK), biochar with chemical fertilizer (CNPK) and straw with chemical fertilizer (SNPK). The results showed that compared with the NPK treatment, CNPK and SNPK significantly increased soil total porosity, soil air permeability coefficient, soil organic carbon (SOC), C/N ratio, soil microbial biomass carbon (SMBC)‚ soil microbial biomass nitrogen (SMBN), invertase activity and rice yield. Furthermore, amendment of biochar had a better effect on SOC, C/N ratio, SMBC, and SMBN than that of straw. In addition, SMBC, SOC, and total nitrogen (TN) had significant correlations with soil enzyme activities. Therefore, amendment of biochar with chemical fertilizer is an effective measure to improve rice production and soil quality in the northeast of China.

## Introduction

In China, the annual planting area of crops is about 1348.8 million hectares, the annual yield of which is about 819 million tons, accounting for about 1/3 of the world’s total production^1^. Traditionally, the majority of plant residues, which are removed from the field after harvesting, are usually used as animal feed, biofuel, and biomass^2^. In most cases, the straw is either burned or discarded, resulting in resources wastage and environmental pollution^3,4^. Reasonable utilization of the straw resources is very important for sustainable agricultural production. Although it has become the first choice^5,6^, returning the straws to the soil is very difficult for the case of large-scale paddy fields in the cold region. In recent years, carbonizing straw into biochar has become a new approach of straw utilization. According to some studies, the biochar that is formed by crop straw and chaff under an oxygen-limited condition plays an essential role in enhancing the storage of organic carbon in the soil, reducing emissions of carbon dioxide emissions, and improving soil fertility^7–9^. The effects of directly applying straw into soil or biochar into soil on soil organic carbon, crop yields, and greenhouse gas emissions have been frequently reported^10–12^.

Biochar, a carbon-enriched solid material with high cation exchange capacity (CEC), large porosity, and high surface area, was produced by agricultural waste, animal manure, and industrial wood by-products^12–14^. The aromatic structure of biochar exhibits the characteristics of high resistance to chemical and biological degradation and stability in soil^15^. Because of its high pH value, biochar can increase the soil pH if applied into the soil. In addition, biochar can increase the soil carbon reserves, hold the soil nutrients, build the soil fertility, and increase the crop yield^16–18^. For example, the biochar amendments significantly increased the rice yield by 15.3–44.9% over the chemical fertilizer through increased fertilizer use efficiency^19,20^. After biochar application for four years, total carbon and total nitrogen of the soil increased by 27.6% and 75.6%, respectively, and peanut yield increased by 50.6%^21^.

Alfisols is one of the most primary arable soils and is considered to be essential for crop production in Northeast China^22^. In China, excessive fertilizer application has been a common practice to achieve high crop yield in the past decades, resulting in the degradation of soil and environmental pollution. Although how the amendment of straw or biochar affects both the soil quality and crop production has been reported extensively^23,24^, few reports about how the straw and biochar made from equal amount of straw as experimental material content influence the crop yield and soil physicochemical and biological properties in the cold region have been studied. In addition, the low temperature during winter in this region will reduce the decomposition rate of soil nutrients and soil microbial activity, which differs from that of other regions of China^25,26^. Thus, a deeper insight into the effect of straw/biochar with chemical fertilizer on soil physicochemical and biochemical properties during a long-term field experiment in this region is necessary.

In this study, a five-year field site experiment was designed to investigate how directly applying the rice straws and biochar into the soil influences the microbial biomass carbon and nitrogen, enzyme activity and physicochemical properties under the equal amount of straws, and equal nutrient condition of nitrogen‚ phosphorus and potassium. This study could provide a theoretical basis for applying the rice straw biochar in the current cropland management systems for local farmers in Northeast China.

## Results

### Effects of biochar and straw application on soil physical properties

Continuous application of biochar and straw had a significant effect on the soil physical properties (Table 1, *p* < 0.05). Compared with the CK treatment, total porosity and air permeability coefficient of the soil increased by 26.9% and 70.2% in the C treatment, respectively, and 21.7% and 62.3% in the S treatment, respectively; while bulk density and hardness significantly decreased by 15.9% and 31.1%, 13.5% and 26.4%, respectively. As compared to NPK treatment, total porosity and air permeability coefficient in the CNPK and SNPK treatment significantly increased by 24.6% and 63.5%, 19.2% and 49.4%, respectively, while soil bulk density and soil hardness significantly decreased by 13.9% and 26.7%, 12.6%, and 22.4%, respectively. In addition, for the CNPK/SNPK treatment, the soil bulk density and hardness significantly decreased, but total porosity and air permeability coefficient significantly increased, as compared with the C/S treatment.

**Table 1:** The effects of different fertilization treatments on soil physical properties.

Treatment	Bulk density (g· cm^−3^)	Total porosity (%)	Air permeability coefficient (10^−5^ cm·s^−1^)	Hardness (kpa)
CK	1.33 ± 0.08a	37.83 ± 1.73c	4.79 ± 1.19b	14.65 ± 2.52ab
NPK	1.37 ± 0.09a	35.63 ± 1.53c	4.52 ± 1.21b	15.70 ± 2.64a
C	1.12 ± 0.09b	48.01 ± 5.3a	8.15 ± 1.62a	10.09 ± 2.28c
CNPK	1.18 ± 0.06b	44.41 ± 3.17ab	7.38 ± 1.17a	11.51 ± 2.03bc
S	1.15 ± 0.10b	46.06 ± 4.512ab	7.77 ± 1.66a	10.79 ± 2.45c
SNPK	1.20 ± 0.06b	42.49 ± 3.85b	6.75 ± 0.85a	12.18 ± 2.11bc

^*^Data here are mean ± SE, *n* = 3. Different lowercase letters refer to soil properties are significantly different among different fertilization treatments according to LSD test (*p* < 0.05). The same in Table 2.

### Effects of biochar and straw application on soil chemical properties

Soil chemical properties differed significantly from each other among different fertilization treatments (Table 2, *p* < 0.05). Compared with the CK treatment, SOC, C/N ratio, and pH in the C treatment significantly increased by 15.07%, 11.6%, and 0.23 units, respectively. Moreover, as compared to the NPK treatment, SOC and pH in the CNPK and SNPK treatment increased by 29.2% and 0.53 units, 9.1% and 0.35 units, respectively. Furthermore, SOC and C/N ratio in the C/CNPK treatment were significantly greater than those in the S/SNPK treatment. In addition, SOC in the CNPK treatment was 8.2% greater than that in the C treatment. TN in the SNPK treatment was 9.6% greater than that in the S treatment.

**Table 2:** The effects of different fertilization treatments on soil chemical properties.

Treatment	TC (g·kg^−1^)	TN (g·kg^−1^)	C/N	pH
CK	6.37 ± 0.06 cd	0.79 ± 0.01c	8.04 ± 0.01 b	6.49 ± 0.15 bc
NPK	6.14 ± 0.10 d	0.83 ± 0.02 bc	7.45 ± 0.12 b	6.05 ± 0.15 d
C	7.33 ± 0.08 b	0.82 ± 0.01 bc	8.97 ± 0.24 a	6.72 ± 0.08 a
CNPK	7.93 ± 0.19 a	0.85 ± 0.01 b	9.31 ± 0.26 a	6.58 ± 0.05 ab
S	6.41 ± 0.02 cd	0.83 ± 0.02 bc	7.78 ± 0.19 b	6.33 ± 0.12 c
SNPK	6.70 ± 0.09 c	0.91 ± 0.10 a	7.41 ± 0.17 b	6.40 ± 0.16 bc

### Effects of biochar and straw application on SMBC and SMBN contents

**Figure 1:**
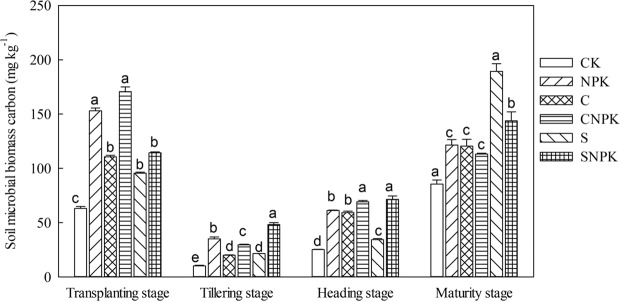
The SMBC of different fertilization treatments at different growth stages. Data here are mean ± SE, *n* = 3. Different lowercase letters indicate the significant difference among different fertilization treatments in the same growth stage according to LSD test (*p* < 0.05). The same in Figs 2–7.

Remarkable differences of SMBC were detected among different treatments at each growth stage (Fig. 1, *p* < 0.05). Compared with the CK treatment, SMBC in the C and S treatments significantly increased at each growth stage. Moreover, compared with the NPK treatment, SMBC in the CNPK treatment increased by 11.6% and 12.9% at the transplanting and heading stages, yet decreased by 15.9% at the tillering stage; SMBC in the SNPK treatment increased by 37.1%, 16.5% and 18.4% at the tillering, heading and maturity stages, respectively, yet decreased by 25.2% at the transplanting stage. In addition, SMBC in the CNPK treatment was significantly lower than that in the SNPK treatment at the tillering and maturity stages, while the opposite trend was exhibited at the transplanting stage. During the whole growth stage, SMBC firstly decreased from transplanting stage to tillering stage, then increased at the heading and maturity stages (Fig. 1, *p* < 0.05).

**Figure 2:**
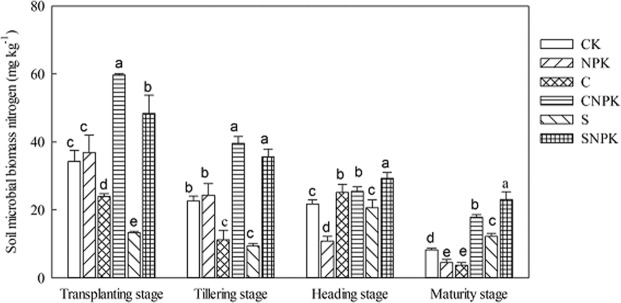
The SMBN of different fertilization treatments at different growth stages.

Obvious differences were also observed in SMBN among different treatments at each growth stage (Fig. 2, *p* < 0.05). Compared with the CK treatment, SMBN in the C treatment decreased by 30.2%, 50.3% and 54.8% at the transplanting, tillering and maturity stages, respectively; SMBN in the S treatment decreased by 61.1% and 58.6% at the transplanting and tillering stages, respectively. Moreover, compared with the NPK treatment, SMBN in the CNPK and SNPK treatments significantly increased at each growth stage. Additionally, SMBN in the CNPK treatment was significantly lower than that in the SNPK treatment at both the heading and maturity stages, while an opposite trend was exhibited at the transplanting stage (Fig. 2, *p* < 0.05). During the whole growth stage, SMBN in CK, NPK, CNPK, and SNPK treatments decreased from transplanting stage to the maturity stage‚ while SMBN in the C and S treatments firstly decreased, then increased, and finally decreased. Compared with transplanting stage, SMBN significantly decreased at the tillering, heading, and maturity stages.

### Effects of biochar and straw application on soil enzyme activities

#### Urease activity

**Figure 3:**
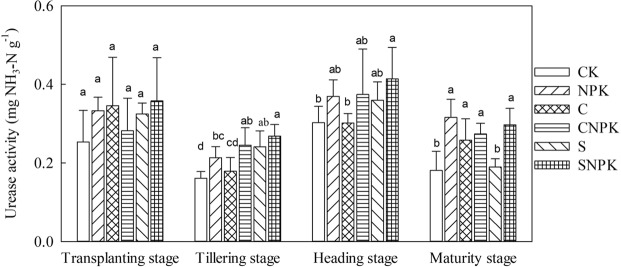
Urease activity in soil under different fertilization treatment at different growth stages.

Remarkable differences were demonstrated in urease activity among different treatments (Fig. 3, *p* < 0.05). Compared with the CK treatment, urease activity in the NPK treatment significantly increased by 32.6% and 74.9% at tillering and maturity stages. In addition, there were no significant differences in urease activity between the CNPK and SNPK, C and CNPK, S and SNPK treatments (Fig. 3, *p* < 0.05). During the whole growth stage, the urease activity firstly decreased from transplanting stage to tillering stage, then increased at the heading stage, and finally decreased at the maturing stage. Compared with transplanting stage, urease activity significantly decreased at the tillering stage for all the treatments.

#### Invertase activity

**Figure 4:**
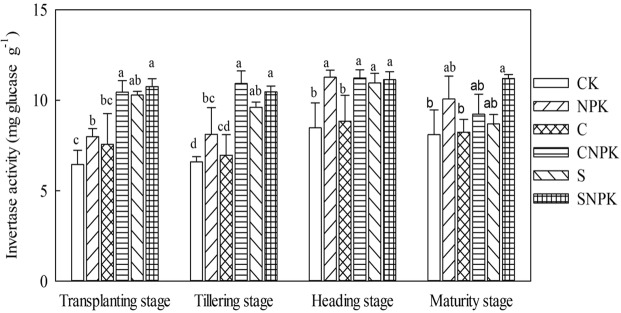
Invertase activity in soil under various fertilization regimens at different growth stages.

The invertase activity exhibited significant differences among different treatments at each growth stage (Fig. 4, *p* < 0.05). Compared with the CK treatment, invertase activity in the NPK treatment significantly increased. Moreover, compared with the NPK treatment, invertase activity in CNPK and SNPK treatments significantly increased by 30.9% and 34.8%, and 34.6% and 29.0% at the transplanting and tillering stages, respectively. In addition, there was no significant difference in invertase activity between C and S, CNPK and SNPK treatments. During the whole growth stage, the invertase activity increased from transplanting stage to heading stage and then decreased at the maturing stage. Compared with transplanting stage, invertase activity increased in the heading stage for all the treatments.

#### Catalase activity

**Figure 5:**
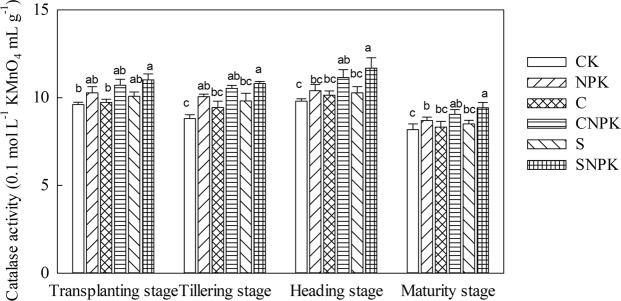
Catalase activity in soil under various fertilization regimens at different growth stages.

Compared with the CK treatment, catalase activity in the NPK treatment significantly decreased (Fig. 5). In addition, there was no significant difference in catalase activity between the CNPK and SNPK treatments. During the whole growth stage, the catalase activity firstly decreased from transplanting stage to tillering stage, then increased at the heading stage, and finally decreased at the maturing stage. Compared with the transplanting stage, catalase activity significantly decreased in the maturity stage for all the treatments.

#### β-glucosidase activity

**Figure 6:**
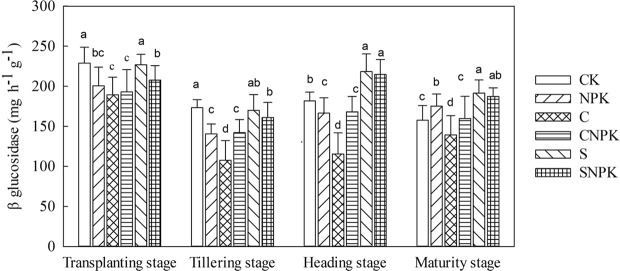
*β*-glucosidase activity in soil under different fertilization treatments at different growth stages.

Compared with the CK treatment, β-glucosidase activity in the NPK treatment significantly decreased (*p* < 0.05, Fig. 6). Moreover, compared with the NPK treatment, β-glucosidase activity in the SNPK treatment significantly increased by 14.6% and 29.0% at the tillering and heading stages, respectively; while that in the CNPK treatment significantly decreased by 8.9% at the maturity stage. Besides, β-glucosidase activity in the CNPK treatment was significantly lower than that in the SNPK treatment. During the whole growth stage, the β-glucosidase activity first decreased from transplanting stage to tillering stage, then increased at the heading stage, and finally decreased at the maturing stage. Compared with transplanting stage, β-glucosidase activity significantly decreased at the tillering, heading, and maturity stages.

### Effects of biochar and straw application on rice yield

**Figure 7:**
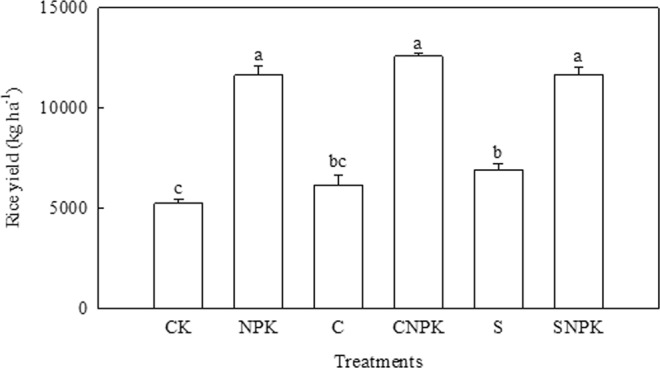
Rice yield response to different fertilization managements.

Different fertilizer management treatments showed different influences on the rice yield (Fig. 7, *p* < 0.05). Compared with the CK treatment, the rice yield in the NPK, C and S treatments increased by 106.9%, 27.8% and 22.2%, respectively. Besides, as compared to the NPK treatment, the rice yield in the CNPK and SNPK treatments increased by 14.5% and 11.8%, respectively. However, no significant difference in the rice yield was shown in both CNPK with SNPK treatments. In addition, as compared with the C and S treatments, the rice yield in CNPK and SNPK treatment significantly increased by 85.4% and 89.4%, respectively.

### Correlation coefficients of soil enzyme activities, soil microbial biomass, soil chemical properties, and rice yield

Urease and invertase activities displayed a similar behavior, exhibiting positive correlations with SMBC and TN but negative correlation with pH (Table 3). However, catalase activity presented the opposite behavior, which had negative correlations with SMBC, SMBN, and TN but positive correlations with pH. The β-glucosidase activities had a negative correlation with SOC and C/N. Rice yield (YIE) had a positive correlation with TN, MBC, MBN, urease activity, and invertase activity but a negative correlation with catalase activity and pH value.

**Table 3:** Correlation coefficient between selected soil chemical properties and biochemical properties.

	URE^†^	INV	HYD	β-GLU	MBC	MBN	SOM	STN	C/N	pH	YIE
URE	1.000**										
INV	0.831**	1.000**									
HYD	−0.837**	−0.658**	1.000**								
β-GLU	−0.005	0.386	0.263	1.000**							
MBC	0.944**	0.869**	−0.849**	−0.049	1.000**						
MBN	0.473	0.606*	−0.530*	0.086	0.414	1.000**					
SOM	0.376	0.335	−0.309	−0.508*	0.466	0.455	1.000**				
STN	0.871**	0.936*	−0.673**	0.288	0.812**	0.762**	0.407	1.000**			
C/N	0.172	0.14	0.14	−0.567*	0.266	0.381	0.976**	0.227	1.000**		
pH	−0.635*	−0.679**	0.865**	−0.092	−0.620*	−0.671**	0.006	−0.657*	0.154	1.000**	
YIE	0.679**	0.654*	−0.850**	0.141	0.616*	0.676**	−0.047	0.699**	−0.202	−0.988**	1.000**

^†^URE, INV, HYD, β-GLU, YIE are represent urease, invertase, hydrogen peroxidase, β-glucosidase, rice yield, respectively.

*significant difference level (*p* < 0.05), **extremely significant level (*p* < 0.01); *n* = 12.

## Discussion

Soil physical quality plays a crucial role in improving the soil chemical and biological environment. The biochar as a soil amendment has been applied to restore eroded or degraded soils^27^. The high porosity, high inner surface area, and large number of micropores of biochar^28,29^ can potentially improve soil physical properties^30^ to create a better environment for the plant root growth and nutrient uptake. Biochar can induce greater decreases in the bulk density, resulting in a great effect on the soil aggregate stability and total porosity^31,32^. Some reports found that biochar added with a rate of 5% (w/w) decreased average pore size in the soil from 0.07 to 0.046 mm^2^ and the soil tensile strength decreased from 466 to 164 kPa^33,34^. In addition, biochar can support the building processes of the soil structure via indirect means, such as providing habitat for soil microorganisms and enzyme activities^30,35,36^. In the present study, both CNPK and SNPK treatments decreased the soil bulk density and soil hardness and increased soil porosity and air permeability coefficient compared with that of the NPK treatment. This indicated that the application of biochar and straw incorporation with NPK are two effective measures to improve the soil physical properties.

Straw plays an important role in SOC sequestration, which is regulated either by the adsorption capacity of organic molecules or occlusion of coarse straw components^37^. Straw return can increase the fractions of labile organic matter^12,38^, showing that SOC significantly increased after short-term wheat/rice straw return. The increase in the soil pH could be explained by the fact that straw contains many alkaline substances^11^. Moreover, biochar may have an inhibitory effect on native SOC decomposition^39^ because it can sequester the carbon^7,40^ and increased the SOC content. However, different responses of biochar and straw application to chemical properties have also been reported in previous studies^38,41^, because of different types of feedstock, production temperature of biochar and straw quality, soil properties, and other environmental parameters^7,8^.

Biochar exhibited a more prominent effect on SOC and the C/N ratio than straw. This indicated that biochar is more beneficial in improving soil chemical properties^7^. This could be explained by the fact that the porous structure, high CEC, and surface area of biochar make it more stable than the easily decomposed straw^42,43^. Additionally, soil nitrogen fixation capacity was also enhanced due to the physical sorption/microbial immobilization capacity of biochar, which led to less available nitrogen, and increased the soil C/N ratio^44^.

Soil microbial biomass, as a living part of soil organic matter (SOM), drives SOM mineralization and nutrient recycling^45^. Both the biochar and straw application affect soil microbial activities through changing the habitats of soil microbes, availability of nutrients, and soil physical properties^46,47^. In the present study, CNPK and SNPK treatments increased SMBC and SMBN as compared with the NPK treatment. This is consistent with the previous finding^1^; indicating that the application of biochar or straw provides carbon and nitrogen resources for the growth and reproduction of soil microorganisms^1^. However, the negative effect of straw application on the SMBC in previous studies was observed^48^, which may be ascribed to that the straw decomposition may have been delayed in autumn and winter, and the growth of soil microorganisms was limited due to lack of available nutrients in spring^45^.

In addition, biochar performed better than straw in SMBC and SMBN. This may be because biochar has much porous structure and higher adsorption capacity for inorganic nutrients than straw, which provides a suitable habitat for soil microorganisms^46,49^. In contrast, some reports found a decrease in microbial activity after biochar application^47,50^. These contrasting results could be related to changes in soil moisture, pH, and nutrient dynamics caused by the chemical components of the straw used.

Compared with transplanting, the content of SMBC and SMBN significantly decreased in other stages. This may be because more soluble organic carbon was accumulated during the freeze-thaw period before transplanting^45^, rice plants need more nutrients from the tillering to the heading stage, then the amount of microbial and soil respiration intensity declines, which in turn decreased the content of SMBC and SMBN. At the maturity stage, the root growth stopped, and the competition effect with soil microorganism decreased, the amount of soil microbial biomass and soil respiration intensity is recovered^46^, so SMBC and SMBN increased. This is conducive to microbial accumulation and soil fertility recovery.

Soil enzyme activity reflects the microbial activity and is sensitive to alterations of the soil conditions. Urease reflects the transformation of soil organic nitrogen into available inorganic nitrogen^50^. In the present study, the application of straw and biochar had no significant effect on urease activity as compared with the CK/CNPK treatment. This was inconsistent with previous reports, in which the application of biochar/straw had a positive or negative effect on the urease activity^51,52^. The possible reason was that the feedstock type, pyrolysis conditions, production method, application rate, and soil types are the governing factors that will influence the nitrogen cycling and urease activity in the soil^53,54^. Compared with transplanting, urease activity significantly decreased in the tillering stage. The possible reason was that the utilization of available inorganic nitrogen reached a maximum in the tillering stage for the application of biochar and straw, and then the urease activity decreased.

Invertase plays an important role in increasing soluble nutrients in the soil, providing sufficient energy for the soil organisms^45^. In the present study, S, SNPK, and CNPK treatments increased the invertase activity as compared with the CK and NPK treatments. However, straw acts significant role in controlling hydrolytic enzyme activities, which are significantly affected by substrate concentrations, and the release of carbohydrate and protein components from straw may stimulate invertase activity^46,52^. Moreover, the application of biochar can increase enzyme activity through increasing the SOM, microbial activity, and microbial biomass or through co-location of enzymes and their interaction with biochar surface^55^. In addition, the opposite results have been reported^46,56^ that the application of biochar decreases invertase activity. This may be attributed to the strong adsorption capacity of biochar and the high pH value caused by biochar addition^12^. Compared with transplanting, urease activity significantly decreased in the heading stage under the C and NPK treatments. This indicated that the urease activity was not sensitive to the growth stage.

Catalase reflects the degree of biological oxidation and microbial activity in the soil and plays an important role in the oxidation of organic matter and humus formation^55^. In the present study, the application of biochar and straw had no significant effect on catalase activity as compared with the CK and NPK treatments. This was different from the reports that biochar application may increase catalase activity due to the enzymatic reactions between biochar and the target substrate and dramatic changes in the biochemical composition of biochar^57^. Compared with transplanting, catalase activity significantly decreased in the maturity stage, except for the C and SNPK treatments. This indicated that catalase activity was at the lowest in the maturity stage, which may be due to the lowest availability of sources at the end of the whole growth stages^58^.

β-glucosidase is an extracellular enzyme involved in carbon mineralization^59^ and functions in maintaining the carbon cycle and nutrient cycle^51^. In the present study, the C/CNPK treatment had a significant increase in β-glucosidase activity as compared with the CK/NPK treatment, but straw application significantly decreased β-glucosidase activity as compared with the CK treatment. This was different from the findings that straw can control the hydrolytic enzyme activity and the release of protein components will enhance the activity of β-glucosidase^52^. Moreover, other studies also reported that the application of biochar with/without chemical fertilizer had a negative^51,60^ or no effect on β-glucosidase activity^53^. This may be due to the strong adsorptive capacity and that biochar exerted a complex effect on soil β-glucosidase activity. Compared with transplanting, β-glucosidase activity significantly decreased in the tillering, heading, and maturity stages. This indicated that soluble organic carbon was highest during transplanting due to the accumulation during the freeze-thaw period^45^; with the rice growth, the content of soluble organic carbon decreased, which will inhibit the β-glucosidase activity.

In general, straw exhibited a more prominent effect on invertase activity and β-glucosidase activity than biochar. This may be because that the rich functional groups and strong adsorption ability of the biochar can make combination between the biochar and the invertase and β-glucosidase, which will inhibit the activity of invertase and β-glucosidase^61^. Moreover, the application of biochar alters the soil pH value greater than that in straw, which may affect the soil environment of invertase activity and β-glucosidase activity. Additionally, straw could effectively improve soil aggregate structure and soil microaggregate stability, thus improving the soil environment and promoting enzyme activity^61^. In addition, biochar with chemical fertilizer performed better in the invertase activity and β-glucosidase activity than sole application of straw. This may be because the biochar intrinsic nutrient was unavailable for plant growth, and the combination of biochar with chemical fertilizer can partly offset the adsorption effect of biochar.

The crop yield can comprehensively reflect the soil fertility. Biochar application improved soil properties, soil nutrient status, and root growth environment. Many reports showed that both the straw and biochar had a good effect on crop yield^11,62^. However, inconsistent effects were also found in previous studies^15,63^. Some studies discovered that straw application decreased the rice yield due to different application methods, amount of straw, and the time of application^5,15^. Although biochar acts as the sink and the source of most available nutrients for the plant growth and yield, there was no significant difference in rice yield between biochar and straw return. In the present study, CNPK and SNPK showed a significant effect on rice yield over NPK. The biochar was a little better than straw incorporation in rice yield, but no significant difference was observed. Because the soil is relatively low in fertility, coarse in texture, and low in pH, and the biochar is alkaline and fine in texture, so it plays an important role in its soil physicochemical and biological properties as well as rice yield. The results are consistent with some previous reports^14,63^.

In China, excessive fertilizer application has been a common practice to achieve high crop yield in the past decades, resulting in the degradation of soil and environmental pollution. The annual pure application rate of chemical fertilizer in China was 6.02 million tons, which was greater than that in the word^1^. Ministry of Agriculture in China has proposed the plan named as “zero increment in chemical fertilizer until 2020” to reduced the utilization of chemical fertilizer. Therefore, the high efficiency of fertilizer and nutrient replace has become the choices^64^. Besides, the National Key Research and Development Project of China on the chemical fertilizer reduction were launched in 2016. The government hopes these projects can lead the farmer to reduce chemical fertilizer utilization. In the present study, amendment of biochar with chemical fertilizer and amendment of straw with chemical fertilizer were two ways to reduce the utilization of chemical fertilizer. The rice yield in these ways was not reduced as compared with chemical fertilizer. Generally, straw return requires lots of manpower and machinery to cooperate, which limit its use. Based on the sustaining soil carbon content and avoiding environmental pollution, biochar has become a new approach of straw utilization^65^. The results of the present study indicated that chemical fertilizer integrating with biochar application may be a good way for chemical fertilizer reduction.

## Materials and Methods

### Experimental site

The study was conducted in the long-term positioning test station at Shenyang Agricultural University (40°48′N, 123°33′E), located in the center of the South Songliao Plain of the northeast China. The study area exhibited a semi-humid, temperate, and monsoon climate. The mean annual temperature was 7.5 °C, annual precipitation was 736.0 mm, and the frost-free period was ranged from 148 to 180 days. The soil in this area was classified as an Alfisol (USDA Taxonomy), and one of the main cultivated soil types in Northeast China. In the soil of the tillage layer (0–20 cm), the organic matter content was 16.2 g kg^−1^; the total N, P, and K contents were 0.90, 0.62, and 18.1 g kg^−1^, respectively; the available N, P, and K were 86.5, 11.6, and 115.0 mg kg^−1^, respectively; and the pH was 6.05. In this experimental field, the continuous rice cropping system has been practiced since 2014.

### Experimental design

The fixed-site field experiment began in 2013. There were six treatments: (i) no amendment of biochar and straw and no fertilization (CK); (ii) chemical fertilizers (NPK); (iii) amendment of biochar only (C); (iv) amendment of biochar with chemical fertilizer (CNPK); (v) amendment of straw only (S); and (vi) amendment of straw with chemical fertilizer (SNPK). For the treatments of NPK, CNPK and SNPK, the contents of N, P, and K was equal. Each treatment had three replicates with a complete randomized design. Each plot was 4 m^2^ (2 m × 2 m) and equipped with artificial penetration filters to avoid a possible surface runoff.

The rice straw collected from the experimental field was cut into a length of about 15 centimeters for use after drying at 60 °C. The annual decomposition rate of straw return in the study area was 4500 kg ha^−1^ a^−1^ ^66,67^. The yield of biochar produced by rice straw at 450 °C for 6 h in Shenyang Agricultural University is about 1:3. The straw amount used for the biochar preparation was the same with that directly applied into the soil. Thus, to keep the same amount of straw return, the application rate of biochar and straw was set as 1500 kg ha^−1^ a^−1^ and 4500 kg ha^−1^ a^−1^, respectively. The properties of biochar and straw are shown in Table 4.

**Table 4:** Essential physical and chemical characteristics of biochar and rice straw.

Materials	Total N (N g kg^−1^)	Total P (P g kg^−1^)	Total K (K g kg^−1^)	Total C (C g kg^−1^)	Specific surface area (m^2^·g^−1^)	Porosity (cm^3^·g^−1^)	Pore diameter (nm)	pH
Biochar	6.48	9.75	15.07	623.5	34.69	0.023	17.12	8.68
Straw	7.06	3.22	10.00	382.3				7.12

*The rice cultivar of Shennong 265 (*Oryza sativa* L. subsp. Japonica cv.) was used in this study. Rice seedlings were cultivated in greenhouse on March 25, and then transplanted into the field on May 10. The transplanting density was 30 cm × 15 cm for each hole, with three seedlings in each hole.

For the NPK treatment, the contents of N, P, and K were 240, 55, and 100 kg ha^−1^, respectively. Besides, for the CNPK and SNPK treatments, the content of N, P and K in biochar and straw were measured before application. The amount of N, P and K in chemical fertilizer needed to integrating with straw or biochar was calculated according the content of N, P, and K in NPK treatment. The application rate of N, P, and K under different fertilization treatments in 2018 is shown in Table 5.

**Table 5:** The application rate of N, P, and K under different fertilization treatments in 2018.

Treatment	Straw kg·ha^−1^	Biochar kg·ha^−1^	N kg·ha^−1^	P kg·ha^−1^	K kg·ha^−1^
NPK	0	0	240	55	100
C	0	1500	0	0	0
CNPK	0	1500	230	40	77
S	4500	0	0	0	0
SNPK	4500	0	210	40	65

For the chemical fertilizer, phosphorus and potassium fertilizers were applied as basal fertilization during transplanting. One-third of the total nitrogen fertilizer was applied basally during transplanting, one-third at the tillering stage, and the remainder as topdressing at the heading stage. Besides, straw and biochar was firstly spread, and then thoroughly mixed with the topsoil (0–20 cm) by plowing one week later after rice harvesting in Autumn.

### Soil sampling

An *in situ* sampling method was used for the analysis of soil physical properties using a soil sampler (DIK) fitted with a 100 cm^3^ ring cutter and was repeated three times per cell. The soil samples were collected from the horizon (0–20 cm) of the experimental site in spring of 2014 and autumn of 2014–2018. Soil samples (0–20 cm) were randomly collected from three sites in each plot using a soil auger (STEPS-42101, Germany) across all of the whole growth stages, including transplanting (10th May), the tillering stage (20th June), heading stage (25th July), and maturity stage (20th September). Samples were sealed in plastic bags, stored on ice in cardboard boxes, and carried back to the laboratory. In the laboratory, parts of the soil samples were air dried and then ground to pass through a 2-mm sieve for chemical properties analysis and the remainder was stored at 4 °C for the determination of soil enzyme activity, soil microbial biomass carbon (SMBC), and soil microbial biomass nitrogen (SMBN).

#### Determination of soil physical properties

Soil bulk density was measured by a drying method; soil hardness was directly measured by a penetration durometer in the field (CP40-2, Australia); soil three-phase ratio was determined by a soil three-phase tester (DIK-1130)^68^; the aeration coefficient was determined by a soil aerometer (DIK-5001)^69^.

#### Determination of soil chemical properties and microbial biomass

Total soil organic carbon (SOC) and total nitrogen (TN) were determined using an elemental analyzer (Vario EL III, Germany). Soil pH was determined in a 1:2.5 soil/water suspension using a pH meter. The SMBC and SMBN were determined by a chloroform-fumigation-extraction method^70^.

#### Determination of soil enzyme activities

Soil urease activity, invertase activity, catalase activity and β-glucosidase activity were all determined by using the corresponding kit produced by Solarbio company^71^. The stopping procedure was operated according to the product manual provided by Solarbio company and determined by iMark microplate reader. Each sample was repeated three times.

### Data analysis

The software SPSS 21.0 (SPSS Inc.) was used to examine significant differences of soil chemical properties, soil microbial biomass, soil enzyme activities, and rice yield among different treatments by one-way analysis of variance (ANOVA). A correlation matrix of the Pearson correlation coefficient was used to analyze the correlations among soil enzyme activities, soil microbial biomass, soil chemical properties, and rice yield.

## Conclusions

Five years continuous application of biochar and straw significantly affected soil physicochemical and biological properties as well as rice yield. Compared with the NPK treatment, CNPK and SNPK significantly decreased soil bulk density, hardness, and increased air permeability coefficient, SOC, pH, SMBC, SMBN, invertase activity and rice yield. In addition, C had a better effect on SOC, C/N ratio, SMBC, and SMBN than S. CNPK and SNPK were much better than C and S on soil physicochemical‚ biological properties and rice yield. Therefore, to prevent soil degradation caused by the abundant use of chemical fertilizers, biochar and straw with chemical fertilizer are two effective measures for the rice production and soil quality improvement in northeastern China, especially for the formers.

## Data Availability

The original data can be obtained from the authors upon reasonable request.
